# Waste Point Identification of Frying Oil Based on Gas Chromatography–Ion Mobility Spectrometry (GC-IMS)

**DOI:** 10.3390/molecules29163979

**Published:** 2024-08-22

**Authors:** Lin Ye, Lijun Song, Li Zhang, Ruiguo Cui

**Affiliations:** 1 College of Food Science and Engineering, Tarim University, Alar 843300, China; yelin1998@163.com; 2 Construction Corps Key Laboratory of Special Agricultural Products Further Processing in Southern Xinjiang, Alar 843300, China; 3 School of Food Science and Technology, Hebei Normal University of Science & Technology, Qinhuangdao 066600, China; slj176@163.com (L.S.); cxbh1984@163.com (L.Z.)

**Keywords:** cottonseed oil, volatile substances, waste indicator, gas phase-ion mobility spectroscopy, waste point

## Abstract

This study described the quality detection and rapid identification of frying oil waste points based on gas chromatography–ion mobility spectrometry (GC-IMS). A total of 48 volatile substances were identified, among which the levels of 11 components, including 2-pentylfuran, 2-butylfuran, and 2-hexanone, increased with prolonged frying time after 40 h in cottonseed oil. Conversely, the levels of hexanal, heptanal, and E,E-2,4-heptadienal decreased as frying time extended. Correlation analysis revealed a significant association between volatile substances of the oil and acid value (*p* < 0.05) and polar components with volatile substances (*p* < 0.05). Furthermore, significant differences in the types and contents of flavor substances were observed in cottonseed oil at different frying times (including before and after reaching the discard point) (*p* < 0.05). Subsequently, principal component analysis (PCA) results clearly showed that the cottonseed oil samples at different frying times were well distinguished by the volatile compounds; moreover, discriminant model analysis indicated a model accuracy rate of 100%. These results showed the potential of GC-IMS-based approaches in discriminating the waste points of frying oil.

## 1. Introduction

Edible oil is commonly utilized for frying [1]. The high temperature required during the frying process results in oxidation in the oil and a series of changes in some composition of frying oil with the extension of frying time, leading to the decline in oil quality [2,3]. Consequently, the quality of fried food also deteriorates. Due to the long-term high-temperature heating of oils and fats, a large number of fatty acids and volatile substances such as aldehydes, ketones, lactones, and others are produced, which pose a serious threat to individuals’ physical and mental well-beings [4,5]. Therefore, robust stability and anoxidation are crucial for edible oil. Furthermore, the distinctive flavor found in fried food originates from unsaturated fatty acids present in frying oil. These unsaturated fatty acids are prone to oxidation at high temperatures. The organic compounds produced through the oxidation of unsaturated fatty acids contribute to this flavor profile by generating ketones and aldehydes. Husked cottonseed contains approximately 40% oil content [6]. As one of the daily edible oils, cottonseed oil plays an important role in the food industry. And its composition of fatty acid and glyceryl ester in cottonseed oil have good crisp performance and natural antioxidant capacity. Therefore, cottonseed oil possesses substantial development potential and serves as a notable choice for frying purposes [7,8]. Therefore, cottonseed oil was used as a representative research object matter in this study.

Recently, various strategies have been developed to distinguish the waste point of oil, including electronic nose and electronic tongue technology [9,10,11], dielectric constant method [12], LF-NMR (low-field nuclear magnetic resonance) [13], TLC (thin-layer chromatography) [14], and IR (infrared spectra) [15,16,17,18]. These conventional analytical methods have high sensitivity and good repeatability. However, they require cumbersome sample processing and relatively expensive reagents, which limit their application for the rapid determination of the waste point of oil. In recent years, with the increasing consumption of fried food in Xinjiang, China, the reuse of frying oil has become a common phenomenon in the market. This has raised concerns about food safety among consumers. Due to numerous and scattered processing enterprises and vendors, market supervision is extremely difficult as there are limited numbers of inspectors available. Therefore, there is a need for rapid determination methods for waste index and the identification of waste points to ensure quality supervision and food safety control.

Gas chromatography-ion mobility spectrometry (GC-IMS) is an emerging technique. Its application in food quality and safety has increased in recent years due to its multiple advantages (e.g., comparatively simple system setups and robustness, rapid identification, sensitiveness, and convenience) over other conventional analytical techniques [19]. It is used to determine volatile compounds in a sample for rapid sample quality detection [20]. Currently, the analysis and detection of GC-IMS in the adulteration and doping of edible oil are at a preliminary stage, with few studies on the judgment of the waste index of frying oil [18,21]. Garrido-Delgado et al. [21] used GC-IMS to establish a discriminant model for olive oil and obtained accurate results. A method of adulteration detection and predictive analysis model with PLS was established by GC-IMS in canola oil. These studies provide the basis for the application of GC-IMS in food analysis and detection. To the best of our knowledge, no attempt has been made to examine the potential use of GC-IMS to detect waste-point oil by detecting the volatile components of frying oil at different frying times.

The present study aimed to establish an emerging method for rapidly identifying waste points in cottonseed oil. Deep-fried noodle cake was used as a frying food and fried at 180 °C for 80 h in cottonseed oil. The changes in the content and composition of volatile substances in cottonseed oil at different frying times were analyzed using GC-IMS, and a fingerprint spectrum database of cottonseed oil was established to provide technical support for the quality detection and rapid identification of waste point in frying oil.

## 2. Results and Discussion

**Figure 1:**
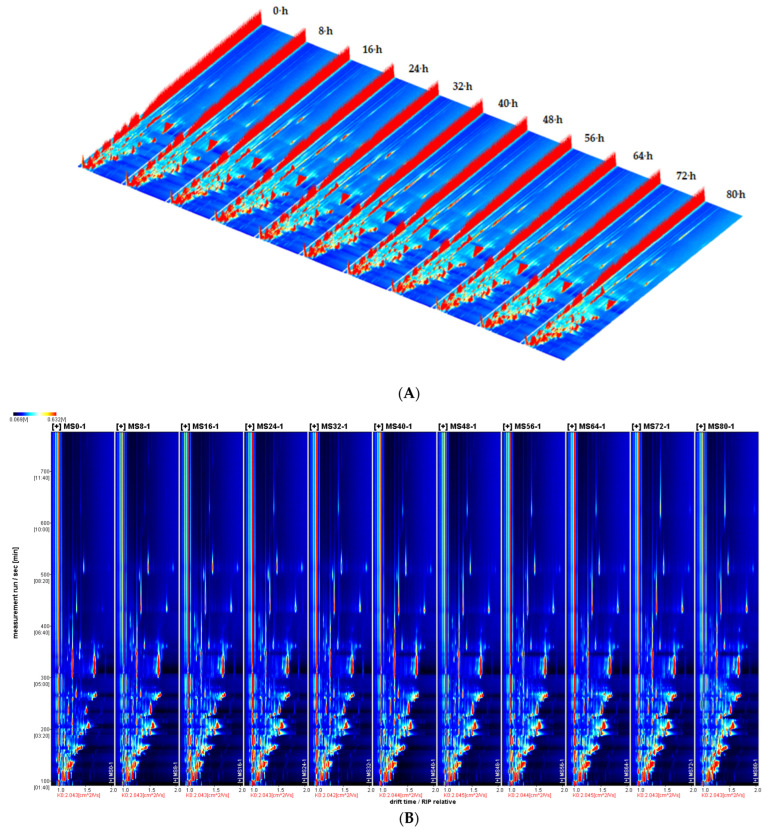

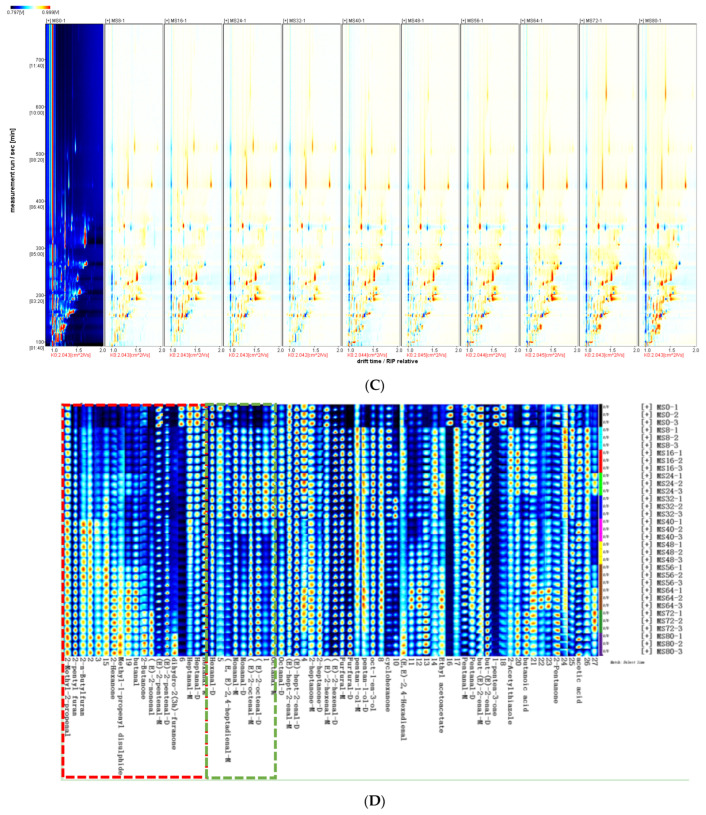
The abbreviations MS0, MS8, MS16, MS24, MS32, MS40, MS48, MS56, MS64, MS72, and MS80 represent 0, 8, 16, 24, 32, 40, 48, 56, 64, 72, and 80 h. (**A**) Three-dimensional topographic plots of volatile compounds in cottonseed oil during frying. (**B**) Two-dimensional topographic plots of volatile compounds during frying in cottonseed oil (top view). (**C**) Comparison and difference spectrum of volatile substances in cottonseed oil during frying. (**D**) Fingerprint of volatile compounds in cottonseed oil during frying.

**Figure 2:**
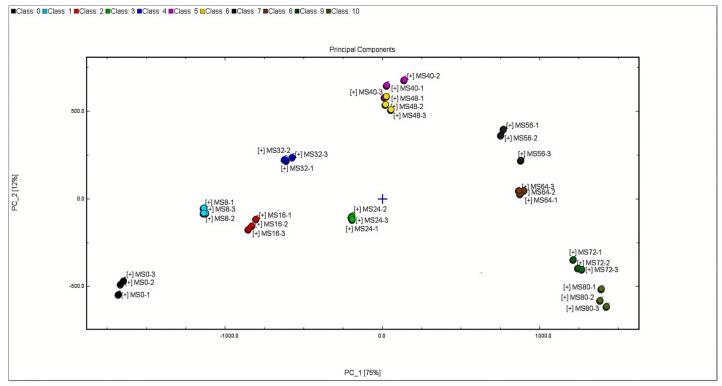
PCA of volatile compounds in cottonseed oil during frying.

The GC-IMS instrument automatically generated 3D topographic plots (Figure 1A) and 2D topographic plots (Figure 1B), illustrating the volatile compound profiles in cottonseed oil during frying. Although the topographic plots exhibited a tendency of volatile compounds, accurately discerning the dense material on the map (Figure 1B,C) proved to be challenging.

### 2.1. Volatile Mass Spectrogram and Fingerprinting of Volatile Compounds in Cottonseed Oil during Frying

The complex composition of volatile compounds is indicated by the 3D topographic plots and 2D topographic plots of GC-IMS spectra shown in Figure 1. The vertical coordinate represented the retention time of gas chromatography(s), while the horizontal coordinate represented the ion migration time (a.u.). The red vertical line denotes the ion reaction peak (RIP peak), with each point on both sides representing a volatile compound [22]. From analyzing the 3D topographic plots, it can be observed that different constituents of volatile compounds are present in cottonseed oil at different frying times. Figure 1B provides a top view of the 3D topographic projected onto a 2D topographic plane. By examining the color intensity of the RIP peak, differences in composition and the concentration difference between volatile compounds in cottonseed oil at different frying times can be intuitively displayed through the 2D topographic (Figure 1B) plots.

The composition and content (Figure 1C) of volatile substances in cottonseed oil were compared at different frying durations. The red color indicated a higher concentration of volatile compounds in the oil sample during frying compared to the reference sample, while blue indicated a lower concentration [23]. It is evident that the red color is obviously darker and gradually increased, with the extension of frying time, indicating an augmentation in both the composition and content of volatile compounds. When the frying duration exceeded 40 h, the concentration of volatile substances showed significant changes, and some volatile flavor substances showed a gradual increase. Therefore, disparities in the composition and content of volatile substances can serve as indicators for determining changes in oil quality.

The results of the qualitative analysis of volatile compounds in cottonseed oil at different frying times are listed in Table 1. A total of 48 monomers and partial dimers were identified from the GC × IMS library, including 16 aldehydes, 7 ketones, 3 acids, 2 alcohols, 2 esters, 2 furans, 1 thiazole, and 1 sulfur-containing compound. Aldehydes were abundant, followed by ketones and acids. The drift time and retention time of volatile compounds were qualitatively analyzed for cottonseed oil at different frying times.

Utilizing fingerprints provided an effective approach to address this issue [24,25]. The horizontal coordinate in the figure (Figure 1D) represented the detected volatile substances, while the numbers indicated undetected substances. The vertical coordinate denoted different frying times for oil samples. Each cell represented the content of a substance at a specific frying time. Colors represented the contents of volatile compounds: the brighter colors indicated higher content [26]. The results revealed that both changes in volatile substances and composition of volatile flavor substances were associated with color brightness. With the extension of frying time, both the variety and concentration of volatile flavor substances in cottonseed oil increased, which may be attributed to the oxidation and cracking of oil during frying. As shown in the red box, after 40 h of frying, significant increases were observed in concentrations of 11 identified components, including 2-methyl-2-acrolein, 2-amylfuran, 2-butylfuran, 2-hexanone, 1-propenylmethyldisulfide, butyraldehyde, 2-butanone, E-2-nonenal, E-2-pentenal monomer, E-2-pentenal dimer, and γ-butyrolactone. As shown in the green box, the concentration of hexal, heptadienal, E, e-2, 4-heptadienal decreased.

In short, the characteristic volatiles fingerprints of cottonseed oil at different frying times were successfully established through GC-IMS, and the changing regular of volatile compounds in cottonseed oil was observed.

### 2.2. Principal Component Analysis (PCA) of Various Cottonseed Oil Samples at Different Frying Times

The PCA is a multivariate statistical analysis method that reveals differences between various samples, with similar samples indicating minimal disparities. Conversely, greater distances on the diagram indicate significant variations in composition [27]. Generally, when the cumulative contribution rate reaches 60%, the PCA model is selected as the separation model [28]. The results of PCA clearly demonstrated that the accumulative variance contribution rate of the first PC (75%) and second PC (12%) amounts to 87%. It is evident that PCA retains the primary information of volatile substances and the characteristics of volatile flavor in each sample. There was a substantial distance between the principal component of cottonseed oil sample at frying 0 h and after frying, indicating significant differences in volatile components before and after frying. From observation, parallel samples cluster closely while non-parallel samples exhibit a certain distance larger than that between parallel ones.

In Figure 2, a distinct separation trend in cottonseed oil samples in volatile components at different frying times can be observed. The cottonseed oil samples fried for 40 h and 48 h exhibit closer proximity, indicating similarity in the volatile compounds during these two periods. On the axis, a large distance was found between the cottonseed oil sample at frying 48 h and 56 h. The samples fried for 56 h and 64 h are easily distinguishable, suggesting a notable difference in the composition and concentration of volatile substances. This implies that the quality of cottonseed oil further deteriorated after frying for 48 h. The results demonstrate that GC-IMS technology can effectively identify and differentiate oils with varying frying times, which aligns with the conclusions drawn in other researchers’ studies. Su [29] conducted principal component analysis on flavor substances during brown rice storage using GC-IMS technology. The results revealed an intensified deterioration in brown rice quality within 70 days at 35 °C and within 28 days at 45 °C, providing a methodological basis for rapid monitoring of brown rice quality. Dirinck [30] analyzed the characteristic flavor components of ginger from three different origins using gas-phase ion migration spectrometry technology. The findings indicated significant differentiation among gingers from various origins, demonstrating that GC-IMS technology can be utilized for origin differentiation, traceability, and quality evaluation.

### 2.3. Correlation Analysis of Waste Index and Volatile Substances in Cottonseed Oil during Frying

Acid value refers to the amount of potassium hydroxide required to neutralize 1 g of free fatty acids [31,32], which is a crucial indicator for measuring the rancidity and deterioration of oils and fats. During frying, oil undergoes hydrolysis, resulting in the formation of free fatty acids that contribute to an increase in acid value [33]. The acid value of cottonseed oil undergoes changes during the frying process of three different types of food at a temperature of 180 °C. As depicted in Figure S1, it can be observed that the acid values of the oils from all three fried foods exhibit a gradual increase with prolonged frying time. Notably, the oil’s acid value shows the most rapid rise during the frying process and reaches 5.64 mg KOH/g at 64 h, indicating that it becomes unfit for further use between 56 and 64 h. Additionally, unsaturated fatty acids are oxidized and hydrolyzed, leading to the decomposition of hydroperoxides and the production of aldehydes in an unstable manner. These aldehydes are subsequently oxidized, causing an elevation in acid value due to acid production [34,35]. Toth [36] discovered that the rise in acid value was mainly attributed totriglyceride oxidation into free fatty acids. Lin [37] demonstrated that the acid value of palm oil increased with prolonged frying time. The correlation between acid value and volatile substances (Table 2) during frying was observed. Notably, there was a significant positive correlation between the acid value and the contents of some volatile substances (*p* < 0.05), such as isobutenol, 2-amylfuran, 2-butylfuran, 2-hexanone, 1-propenyl methyl disulfide, butanal, butanone, E-2-nonenal, E-2-pentenal dimer. These findings suggest that volatile components present in cottonseed oil can serve as a rapid determination method for assessing its acid value.

Polar compounds (PCs) were the result of greater polarity in compounds than triglycerides, caused by the oxidation, polymerization, and pyrolysis of oils fried at high temperatures [38]. The detection of polar components is important as it contains almost all products resulting from the oxidation, polymerization, hydrolysis, and pyrolysis of frying oil [39]. The content of the polar components is positively correlated with frying time and the increase in frying temperature for different oils [40]. As shown in Figure 1D, with the extension of frying time, both the variety and concentration of volatile flavor substances in cottonseed oil increased, which may be attributed to the oxidation and cracking of oil during frying. PCs and volatile substances (Table 3) showed correlation during frying. There was a significant positive correlation between the PC and the contents of some volatile substances (*p* < 0.05), such as isobutenol, 2-amylfuran, 2-butylfuran, 2-hexanone, 1-propenyl methyl disulfide, butanal, butanone, E-2-nonenal, E-2-pentenal dimer. The results indicated that volatile components in cottonseed oil can be used for the rapid determination of PC.

### 2.4. Support Vector Machine (SVM) Model Analysis Diagram

**Figure 3:**
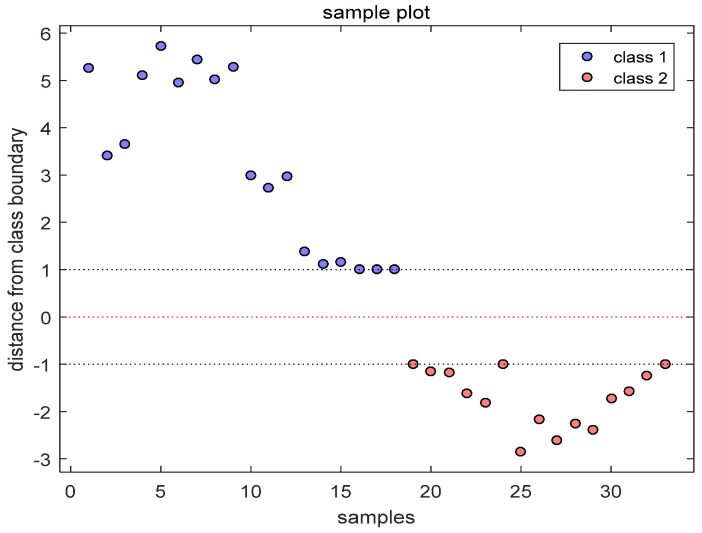
Support vector machine model analysis diagram of naan before and after being discarded in the process of frying in cottonseed oil; class1 and class2 present naan before and after being discarded in the process of frying in cottonseed oil.

The volatile compounds of naan during frying were predicted using a support vector machine (SVM) in Matlab 2018a, (version 3.23) and a discriminative model for distinguishing cottonseed oil was established. Cottonseed oil samples before and after discarding were classified as class1 and class2 (Figure 3). The findings indicated significant differences in the volatile compounds of oil before and after waste treatment. The model achieved an accuracy rate of 100%, indicating a clear differentiation between the volatile compounds of cottonseed oil before and after discarding. Tata [41] utilized GC-IMS technology to detect soft-refined oils in extra virgin olive oil, establishing an SVM mode with an accuracy rate of 100.0%. This study also verified that GC-IMS technology can rapidly identify the point at which frying oil becomes waste.

## 3. Materials and Methods

### 3.1. Materials

Deep-fried noodle cake used in the experiment was obtained from our own laboratory. Samples (100 mL) of cottonseed oil were withdrawn at selected times during the deep frying of the deep-fried noodles at 180 °C. Cottonseed oil was provided by Alar Jinkouxiang Oil Co., Ltd. (Alar, Xinjiang, China). Isopropyl alcohol, ethyl ether, and ethanol were acquired from Shanghai Yuanye Bio-Technology Co., Ltd. (Shanghai, China).

### 3.2. GC-IMS and Acid Value Analysis Method

For GC-IMS analysis, a GC-IMS system (Flavourspec^®^, G.A.S, Dortmund, Germany) equipped with an MXT-5 capillary column (15 m × 0.28 mm ID, 0.25 μm film thickness) was employed in this study, which was used in conjunction with the CTC-PAL·3 Static Headspace Automatic Injection device from CTC Analytics AG in Zwingen, Switzerland. Data processing was performed using the vOCa software (version 0.4.03) also developed by G.A.S.

The oil sample was mixed with 1 mL of *n*-hexane by vortex, and then filtered through membranes (0.22 μm). Then, 1 mL mixtures were placed in a 20 mL headspace glass sampling vial. Subsequently, these samples were incubated at 80 °C for 15 min with speed agitation of 500 rpm. Subsequently, 500 μL of headspace was automatically injected into the splitless injector (80 °C) by means of a heated syringe at 85 °C. The carrier gas (nitrogen of 99.999% purity) passed through the GC-IMS injector transferring the sample into the capillary column at 2 mL min^−1^ over the first 2 min, and then it increased up to 100 mL min^−1^ for 20 min, and the drift gas flowrate in drift tube was 150 mL/min. The resulting ions were driven to the drift tube which operated on a constant temperature (45 °C).

Acid-base titration is a classical technique employed to determine the acid value of edible oils. In this method, the oil sample is initially dissolved in a non-polar organic solvent. Then, an acidic indicator, such as phenol phthalide, is added to facilitate the neutralization reaction between the sodium hydroxide solution and free fatty acids present in the oil. The neutralization point of the acid-base reaction is determined through titration, allowing for the calculation of the acid value.

The acid value is determined by the following calculation:

$$X_{AV}=\frac{(V-V_{0})\times c\times 56.1}{m}$$

*X_AV_*—acid value, mg/g;

*V*—the amount of the standard titration solution consumed by the sample is quantified, mL;

*V*_0_—the determination of the corresponding blank for the consumed volume of the standard titration solution, mL;

*c*—the concentration of the titration solution in terms of molarity, mol/L;

### 3.3. Data Processing and Statistical Analysis

The qualitative analysis of the flavor substances in oil sample was performed using the NIST and IMS databases. Reporter and Gallery Plot was applied to record the fingerprints and difference profiles of volatile components. Principal component analysis was performed by Dynamic PCA. The discriminant model was established by matlab2018a software. Experimental data were analyzed statistically using SPSS 25.0, with mean separation at *p* < 0.05 level of significance.

## 4. Conclusions

In this study, 48 volatile compounds were identified via GC-IMS in cottonseed oil at different frying times. Among the identified substances, 11 volatile substances, including 2-amylfuran, 2-butylfuran, and 2-hexanone in cottonseed oil increased significantly with the extension of frying time, while hexanal, heptanal, E, E-2, 4-heptadienal decreased significantly. In addition, the results of PCA also clearly showed that the different cottonseed oil samples at different frying times were in a relatively independent space and were well distinguished. The correlation analysis showed that the acid value of oil was significantly correlated with volatile substances and polar components with volatile substances (*p* < 0.05). The statistical analysis and difference analysis showed that there were significant differences in volatile substances in frying oil before and after waste. The analysis of discriminant model showed that the accuracy of the established model was 100%, which could distinguish waste point of cottonseed oil. Therefore, this study established a rapid method to distinguish waste point of cottonseed oil by GC-IMS. Moreover, according to the differences in the key volatile compounds in cottonseed oil at different frying times, further refinement research can be combined with the relevant processing technology to produce a specific flavor for further fine research in order to find the best way to distinguish the waste point of cottonseed oil.

## Figures and Tables

**Table 1:** Volatile substances identified during frying in cottonseed oil.

No.	Compounds	Formula	RI	Rt (s)	Dt (RIP Relative)	Comment
1	(E)-non-2-enal	C_9_H_16_O	1193	629	1.4138	
2	Nonyl aldehyde	C_9_H_18_O	1112	512	1.4750	Monomer
3	Nonyl aldehyde	C_9_H_18_O	1111	511	1.9491	Dimer
4	Trans-2-Octen-1-al	C_8_H_14_O	1058	435	1.3391	Monomer
5	Trans-2-Octen-1-al	C_8_H_14_O	1058	434	1.8250	Dimer
6	Octyl aldehyde	C_8_H_16_O	1008	362	1.4036	Monomer
7	Octyl aldehyde	C_8_H_16_O	1007	361	1.8284	Dimer
8	2-pentylfuran	C_9_H_14_O	997	346	1.2560	
9	Trans-2-Heptenal	C_7_H_12_O	956	311	1.2575	Monomer
10	Trans-2-Heptenal	C_7_H_12_O	959	314	1.6738	Dimer
11	Heptaldehyde	C_7_H_14_O	901	264	1.3286	Monomer
12	Heptaldehyde	C_7_H_14_O	902	265	1.7013	Dimer
13	2-Heptanone	C_7_H_14_O	894	258	1.2632	Monomer
14	2-Heptanone	C_7_H_14_O	893	258	1.6372	Dimer
15	Trans-2-hexenal	C_6_H_10_O	849	234	1.1847	Monomer
16	Trans-2-hexenal	C_6_H_10_O	852	235	1.5234	Dimer
17	2-Furaldehyde	C_5_H_4_O_2_	832	224	1.0866	Monomer
18	2-Furaldehyde	C_5_H_4_O_2_	829	223	1.3361	Dimer
19	Hexanal	C_6_H_12_O	795	205	1.2574	Monomer
20	Hexanal	C_6_H_12_O	795	205	1.5651	Dimer
21	1-Pentanol	C_5_H_12_O	769	193	1.2556	Monomer
22	1-Pentanol	C_5_H_12_O	766	192	1.5138	Dimer
23	1-Octen-3-ol	C_8_H_16_O	985	336	1.1616	
24	Trans,trans-2,4-Heptadienal	C_7_H_10_O	1014	371	1.1914	
25	Tetrahydrothiophen-3-one	C_4_H_6_OS	943	300	1.1802	
26	(E)-pent-2-en-1-al	C_5_H_8_O	755	187	1.1076	Monomer
27	(E)-pent-2-en-1-al	C_5_H_8_O	755	187	1.3636	Dimer
28	Trans,trans-2,4-Heptadienal	C_7_H_10_O	1013	369	1.6262	
29	Cyclohexanone	C_6_H_10_O	898	262	1.1523	
30	Hexa-2,4-dienal	C_6_H_8_O	913	274	1.1118	Dimer
31	2-Hexanone	C_6_H_12_O	787	200	1.5056	
32	Ethyl acetoacetate	C_6_H_10_O_3_	917	278	1.1479	
33	Allylmethyl disulphide	C_4_H_8_S_2_	937	295	1.1441	
34	Valeraldehyde	C_5_H_10_O	696	163	1.1830	
35	Valeraldehyde	C_5_H_10_O	699	164	1.4278	
36	Crotonaldehyde	C_4_H_6_O	655	151	1.0363	
37	Crotonaldehyde	C_4_H_6_O	650	150	1.2045	
38	2-Methacrylaldehyde	C_4_H_6_O	583	131	1.2210	
39	Ethyl vinyl ketone	C_5_H_8_O	682	158	1.3167	
40	2-Butanone	C_4_H_8_O	592	134	1.2495	
41	2-Acetylthiazole	C_5_H_5_NOS	1006	360	1.1301	
42	Gamma-butyrolactone	C_4_H_6_O_2_	918	279	1.3050	
43	n-Butyric acid	C_4_H_8_O_2_	817	216	1.1620	
44	Propanoic acid	C_3_H_6_O_2_	684	159	1.2641	
45	2-n-Butylfuran	C_8_H_12_O	894	258	1.1835	
46	2-Pentanone	C_5_H_10_O	693	162	1.3728	
47	Butyraldehyde	C_4_H_8_O	597	135	1.2911	
48	Acetic acid	C_2_H_4_O_2_	593.2	134	1.1543	

RI: the retention index, Rt: the retention time, Dt: the drift time.

**Table 2:** Correlation analysis between acid value and volatile substances of deep-fried noodle cake during frying in cottonseed oil.

	Acid Value	2-Methyl-2-propen-1-ol	Empirical Formula	2-n-Butylfuran	2-Butanone	Allylmethyl Disulphide	Butyraldehyde	2-Butanone	(E)-Non-2-enal	(E)-Pent-2-en-1-al Monomer	(E)-Pent-2-en-1-al Dimer
Acid value	1										
2-Methyl-2-propen-1-ol	0.793 **	1									
Empirical Formula	0.891 **	0.964 **	1								
2-n-Butylfuran	0.858 **	0.962 **	0.970 **	1							
2-Butanone	0.954 **	0.771 **	0.891 **	0.817 **	1						
Allylmethyl disulphide	0.912 **	0.909 **	0.945 **	0.927 **	0.882 **	1					
Allylmethyl disulphide	0.937 **	0.720 *	0.812 **	0.808 **	0.889 **	0.879 **	1				
2-Butanone	0.753 **	0.419	0.541	0.478	0.771 **	0.612 *	0.591	1			
(E)-non-2-enal	0.843 **	0.709 *	0.801 **	0.706 *	0.889 **	0.717 *	0.641 *	0.808 **	1		
(E)-pent-2-en-1-al Monomer	−0.374	−0.099	−0.104	−0.138	−0.184	−0.202	−0.26	−0.535	−0.321	1	
(E)-pent-2-en-1-al Dimer	0.676 *	0.680 *	0.770 **	0.711 *	0.792 **	0.705 *	0.533	0.632 *	0.759 **	0.187	1

Results are presented as mean ± SEM with the level of significance, * *p* < 0.05, ** *p* < 0.01.

**Table 3:** Correlation analysis between polar components and volatile substances of naan during frying in cottonseed oil.

	Polar Components	2-Methyl-2-propen-1-ol	Empirical Formula	2-n-Butylfuran	2-Butanone	Allylmethyl Disulphide	Butyraldehyde	2-Butanone	(E)-Non-2-enal	(E)-Pent-2-en-1-al Monomer	(E)-Pent-2-en-1-al Dimer
Polar components	1										
2-Methyl-2-propen-1-ol	0.849 **	1									
Empirical Formula	0.939 **	0.964 **	1								
2-n-Butylfuran	0.872 **	0.962 **	0.970 **	1							
2-Butanone	0.959 **	0.771 **	0.891 **	0.817 **	1						
Allylmethyl disulphide	0.936 **	0.909 **	0.945 **	0.927 **	0.882 **	1					
Allylmethyl disulphide	0.850 **	0.720 *	0.812 **	0.808 **	0.889 **	0.879 **	1				
2-Butanone	0.741 **	0.419	0.541	0.478	0.771 **	0.612 *	0.591	1			
(E)-non-2-enal	0.880 **	0.709 *	0.801 **	0.706 *	0.889 **	0.717 *	0.641 *	0.808 **	1		
(E)-pent-2-en-1-al Monomer	−0.249	−0.099	−0.104	−0.138	−0.184	−0.202	0−0.26	−0.535	−0.321	1	
(E)-pent-2-en-1-al Dimer	0.797 **	0.680 *	0.770 **	0.711 *	0.792 **	0.705 *	0.533	0.632 *	0.759 **	0.187	1

Results are presented as mean ± SEM with the level of significance, * *p* < 0.05, ** *p* < 0.01.

## Data Availability

The data are contained within the article.

## Supplementary Materials

The following supporting information can be downloaded at: https://www.mdpi.com/article/10.3390/molecules29163979/s1, Figure S1: The acid value variation diagram of cottonseed oil during food frying at different temperatures.
