# Supplementary material for: Waste Point Identification of Frying Oil Based on Gas Chromatography–Ion Mobility Spectrometry (GC-IMS)
Source: Molecules. 2024 Aug 22;29(16):3979. doi: 10.3390/molecules29163979 (PMC11357186; doi:10.3390/molecules29163979)
Supplement: Supplementary file 1 [file molecules-29-03979-s001.zip › molecules-3140145-supplementary.pdf]

Supporting material

Waste Point Identification of Frying Oil Based on Gas Chromatography–Ion Mobility Spectrometry (GC-IMS)

Lin Ye [1,2], Lijun Song [3], Li Zhang [3] and Ruiguo Cui [3,*]

1 College of Food Science and Engineering, Tarim University, Alar 843300, China; yelin1998@163.com
2 Construction Corps Key Laboratory of Special Agricultural Products Further Processing in Southern Xinjiang, Alar 843300, China
3 School of Food Science and Technology, Hebei Normal University of Science & Technology, Qinhuangdao 066600, China; slj176@163.com (L.S.); cxbh1984@163.com (L.Z.)
* Correspondence: ruigc_hnust@163.com

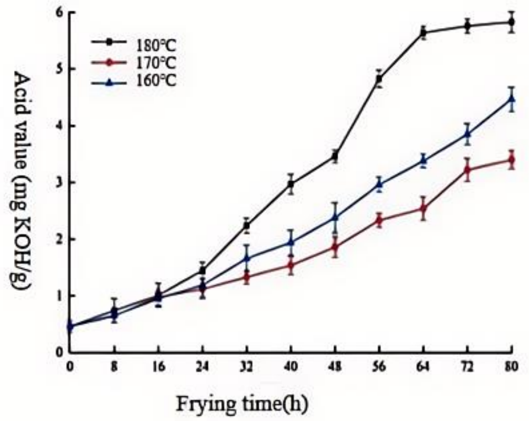

Figure S1. The acid value variation diagram of cottonseed oil during food frying at different temperatures.
